# MRI with hepatobiliary contrast

**DOI:** 10.1186/1470-7330-15-S1-O18

**Published:** 2015-10-02

**Authors:** Kartik S Jhaveri

**Affiliations:** 1 University of Toronto, Abdominal Imaging, University Health Network, Mt.Sinai and WCH 610 University Ave, 3-957, Toronto, ON M5G 2M9, Canada

## 

Hepatobiliary contrast agents currently are essentially gadolinium based agents (Gd-EOB-DTPA and Gd-BOPTA) with dual ability to perform dynamic contrast enhanced imaging similar to extracellular gadolinium contrast as well as providing hepatobiliary uptake and excretion in later phases. Hepatobiliary uptake and excretion with gadoxetic acid (Gd-EOB-DTPA) is related to OATP and cMOAT and MRP2 receptors presence on hepatocytes. Since gadoxetic acid has a recommended dose which is 25 %( 0.025mmol/kg) of Gd-DTPA attention to technical parameters is crucial. Improved arterial phase enhancement is obtained by MR fluoroscopic or bolus-tracking type triggering technique and either a lower injection flow rate of 1 mL/s or less as opposed to 2 mL/s, or contrast dilution. To improve liver-lesion contrast-to-noise ratio, 3D T1-weighted gradient-echo acquisition for the hepatobiliary phase should be performed with higher flip angles (20-35°). In order to optimize workflow, Diffusion-weighted and T2-weighted imaging can be performed after gadoxetic acid administration without compromising diagnostic capability; however, MRCP pulse sequences should be acquired before the contrast injection.

The clinical utilization of Hepatobiliary contrast agents is predominantly for staging of liver metastases, characterization of hepatocellular lesions such as adenoma and FNH as well as diagnosis of HCC and cirrhosis related nodules. In the preoperative setting for accurate evaluation of colorectal liver metastases and appropriate surgical planning, gadoxetic acid-enhanced liver MRI is recommended because it has superior sensitivity and specificity compared to ultrasound, PET, and CT. In the assessment of patients with colorectal liver metastases who have been treated with chemotherapy, preoperative imaging with gadoxetic acid may be of particular benefit. For the differentiation of focal nodular hyperplasia from hepatic adenoma, gadoxetic acid-enhanced MRI should be considered due to its discriminative ability between the two based on hepatobiliary phase features. The combination of hypointensity on hepatobiliary phase images and mild-to-moderate arterial enhancement for adenoma versus strong enhancement on arterial phase images and iso- or hyperintensity on hepatobiliary phase images for FNH showed sensitivity and specificity of 83.7% and 100% and 83.8% and 98.5%, respectively. However a small percentage of adenomas can exhibit hepatobiliary uptake and surveillance and or biopsy should be considered when imaging appearances are not typical. Although gadoxetic acid-enhanced MRI yields significantly higher diagnostic accuracy and sensitivity compared with multiphasic CT for the diagnosis of HCC in cirrhosis, its role in the clinical management of HCC has yet to be defined in North America while it has seen widespread implementation in Asia/Japan. There is insufficient evidence supporting cost-effectiveness or outcomes for recommending the utilization of gadoxetic acid-enhanced MRI for HCC screening at this time. A significant percentage of nodules with hepatobiliary phase hypoenhancement but atypical enhancement on the dynamic phases have been associated with a diagnosis of HCC or future development of HCC. Biopsy or close surveillance of these lesions is recommended. Off label applications include evaluation of biliary disorders, bile leaks and hepatic function. Gadoxetic acid-enhanced liver MRI is an evolving technique with potential for non-invasive quantification of liver function and staging of hepatic fibrosis.
